# A general index for linear and nonlinear correlations for high dimensional genomic data

**DOI:** 10.1186/s12864-020-07246-x

**Published:** 2020-11-30

**Authors:** Zhihao Yao, Jing Zhang, Xiufen Zou

**Affiliations:** 1 School of Mathematics and Statistics, Wuhan University, Wuhan, 430072 China; 2 Hubei Key Laboratory of Computational Science, Wuhan University, Wuhan, 430072 China

**Keywords:** High-dimensional data, Nonlinear correlation, RV-coefficient

## Abstract

**Background:**

With the advance of high throughput sequencing, high-dimensional data are generated. Detecting dependence/correlation between these datasets is becoming one of most important issues in multi-dimensional data integration and co-expression network construction. RNA-sequencing data is widely used to construct gene regulatory networks. Such networks could be more accurate when methylation data, copy number aberration data and other types of data are introduced. Consequently, a general index for detecting relationships between high-dimensional data is indispensable.

**Results:**

We proposed a Kernel-Based RV-coefficient, named KBRV, for testing both linear and nonlinear correlation between two matrices by introducing kernel functions into RV_2_ (the modified RV-coefficient). Permutation test and other validation methods were used on simulated data to test the significance and rationality of KBRV. In order to demonstrate the advantages of KBRV in constructing gene regulatory networks, we applied this index on real datasets (ovarian cancer datasets and exon-level RNA-Seq data in human myeloid differentiation) to illustrate its superiority over vector correlation.

**Conclusions:**

We concluded that KBRV is an efficient index for detecting both linear and nonlinear relationships in high dimensional data. The correlation method for high dimensional data has possible applications in the construction of gene regulatory network.

## Background

**Fig. 1:**
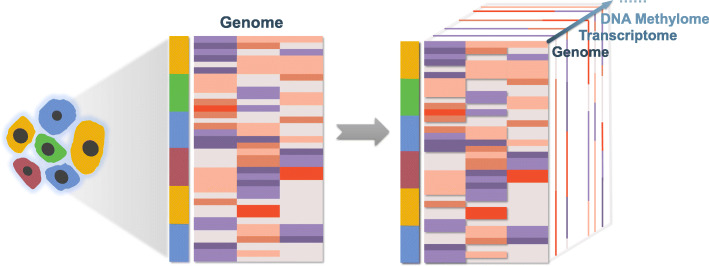
Diversity of cellular data types. Increasing types of single-cell data are available with the advance of sequencing technology, including genome, transcriptome, DNA methylome, etc

With the rapid advance in high throughput sequencing technologies, multiple, high-dimensional data types are widely available. In recent years, the advance in next-generation sequencing and single-cell sequencing offers a significantly increased level of biological details than just total gene expressions [1–5]. Moreover, research based on exon-level expression data, multiomics data and other high-dimensional biological data has led to a deeper understanding of biology [6–8]. Figuratively speaking, the traditional genome data have been extended to transcriptome, DNA methylome data, etc., which reveal overall pictures of cells (Fig. 1). A useful practice in high-dimensional data integration is to measure and rank the dependence between pairs of datasets in a simple and comprehensive way and these datasets are usually represented as matrices or even tensors. Therefore, one of the challenging tasks is how to define a reasonable correlation coefficient between pairs of high-dimensional data sets in matrix form.

**Fig. 2:**
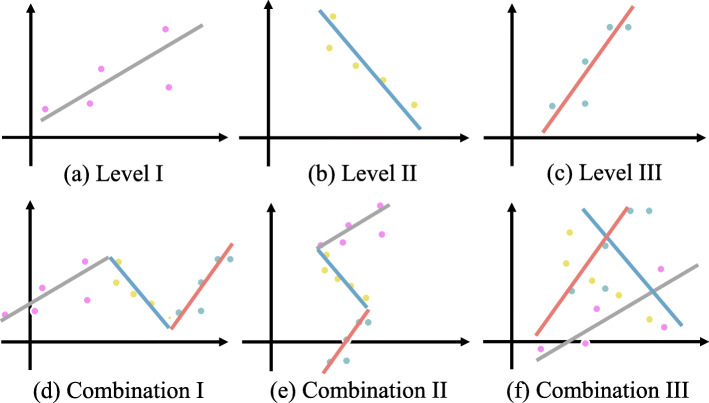
Correlations and their combinations. **a**-**c** Different aspects of correlation show different information. **d**-**f** Different combinations of correlations may have different results, which is misunderstanding and makes it hard for us to evaluate the true relationship

Although a great number of tests and measures are available for identifying linear and nonlinear correlations between two variables, such as Pearson Correlation Coefficient (PCC), Mutual Information (MI) and the Maximal Information Coefficient (MIC), et al. [9–11], it is difficult to evaluate relationships between a pair of matrix. In practice, vector’s correlation methods are widely used in high-dimensional data sets whose mathematical form are matrices, which could lead to wrong results since people just splice matrix into several vectors and ignore the whole structure of the matrix. Normally, the information obtained from data depends on the perspective of the observation. When we observe high-dimensional data *A* and *B*, we might see the data from different aspects and acquire different information. For example, when we look at the expression of gene pairs, if we consider level I, level II and level III in Fig. 2a-c as three different time periods, combination I in Fig. 2d indicates their overall change over time. Besides, level I-III could also be regarded as three samples from different human tissues at the same time so that the combination II described the expression of gene pairs in different tissues. What’s more, if level I, level II and level III represent three different types of omics data observed in the same tissues at the same time, then Fig. 2f could be viewed as a combination of them. The above three examples could be deemed as relationships between genes in the form of two matrices rather than vectors. A simple combination of different information from the perspective of vectors could lead to a misunderstanding and makes it hard for us to evaluate the true relationship. In exploring the correlations between matrices, Robert and Escoufier first proposed the RV-coefficient in multivariate analysis and Ramsay applied it to high-dimensional data [12, 13]. Furthermore, Smilde et al. presented the modified RV-coefficient (RV_2_) to improve this measure and extended it to partial matrix correlations [14, 15]. Recently, Borzou et al. derived RV into three unique parts with different functions [16]. Wang et al. integrated RV and mutual information into Iso-Net for predicting functions of isoforms with the exon-level RNA-Seq data [17]. Though these coefficients are getting better at measuring a linear correlation between two matrices, they lose efficacy when used to detect a nonlinear correlation.

In this work, we propose a novel index for testing the nonlinear dependence between two matrices by introducing kernel functions into RV_2_. We conduct simulation studies to verify the rationality of KBRV and discuss its application using examples with real data.

## Methods

The underlying assumptions is that two high-dimensional datasets could be represented as two matrices $A \in \mathbb {R}^{M\times {N_{1}}}, B \in \mathbb {R}^{M\times {N_{2}}}$, respectively, which indicates that both matrices require an equal number of rows *M*. Previous work has showed that the matrix RV-coefficient was presented to quantitatively evaluate the correlation between any two matrices *A* and *B* sharing the row-mode.

1 $$ RV(A,B) = \frac{tr\left(AA^{T}BB^{T}\right)}{\sqrt{tr\left[\left(AA^{T}\right)^{2}\right]tr\left[\left(BB^{T}\right)^{2}\right]}}  $$

Subsequently, Smilde et al. found RV’s drawbacks in a transcriptomics study, i.e. the RV values were high in almost all cases especially for some strongly unequal sized matrices where the number of rows are much smaller than the number of columns. It was assumed that *A*(*M*×*N*_1_) and *B*(*M*×*N*_2_) are two random matrices, with elements drawn from standard normal distributions and *N*_1_,*N*_2_ much larger than *M*. Based on the expression of RV and some properties of normal distributions, the RV-coefficient can be approximated as

2 $$ RV(A,B) \approx \frac{N_{1}N_{2}}{\sqrt{\left(N_{1}^{2}+(M+1)N_{1}\right)\left(N_{2}^{2}+(M+1)N_{2}\right)}}.  $$

From (2) it can be found that the value of RV is directly associated with the number of rows and columns of the matrices. When *M* is small enough compared to *N*, the value of RV is close to 1. To solve this problem, Smilde et al. proposed a new correlation coefficient, the modified RV-coefficient (RV_2_), by replacing *AA*^*T*^ to $\widetilde {AA^{T}}$ ($\widetilde {AA^{T}}$ means *AA*^*T*^−*diag*(*AA*^*T*^)). Through this method, *N*_1_ and *N*_2_ were eliminated because the information of *N*_1_ and *N*_2_ were contained in the diagonal elements of the matrices. Thus, the modified RV-coefficient can be written as

3 $$ RV_{2}(A,B) = \frac{tr\left(\widetilde{AA^{T}}\widetilde{BB^{T}}\right)}{\sqrt{tr\left[\left(\widetilde{AA^{T}}\right)^{2}\right]tr\left[\left(\widetilde{BB^{T}}\right)^{2}\right]}}.  $$

Compared to the RV-coefficient, RV_2_ not only solves the problem caused by high dimension, but also makes it possible to take negative values.

**Fig. 3:**
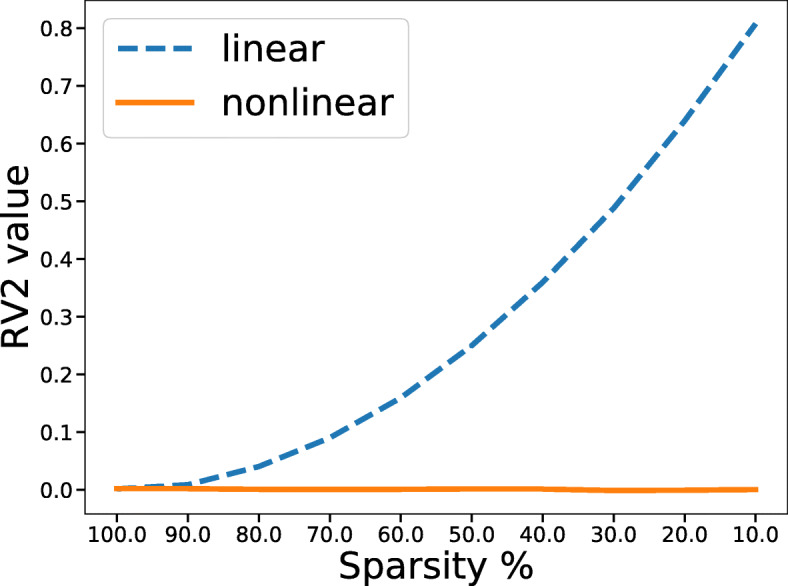
RV_2_ for testing linear and nonlinear correlation. The curve of RV_2_ with an increasing of sparsity between two matrices A and B under linear and nonlinear relationship $\left (b_{ij}=a_{ij}^{2}\right)$

RV_2_ performs well in the linear situation. However, it will have difficulty in detecting nonlinear relationships between matrices. Here we give a simple example. *A*(*a*_*ij*_) and *B*(*b*_*ij*_) are random matrices of size 1000 × 1000 with *a*_*ij*_ and *b*_*ij*_ drawn from standard normal distributions. For the linear situation, we start by calculating *RV*_2_(*A*,*B*). Then, ten percent of the random elements in matrix *B* are replaced by the corresponding elements in matrix *A*. This procedure is repeated with 10 more percent of the elements replaced each time until *A* and *B* are equal. In the other case, an increasing proportion of elements in matrix *B* is replaced by $a_{ij}^{2}$, which indicates a growing nonlinear relationship. That is to say, we want to test the performance of RV_2_ as the linear or nonlinear correlation of *A* and *B* increases step by step. If RV_2_ is effective, the value of RV_2_ would increase in both cases as the proportion grows. The result is shown in Fig. 3, which demonstrates that RV_2_ fails when the correlation is nonlinear.

The nonlinear relationship between matrix *A* and *B* can be generalized as *B*=*f*(*A*)+*noise*, where *f* represents any smooth nonlinear function or transformation and *noise* represents noises with different distributions. In this paper, we mainly discuss the nonlinear *f* with element wise. Therefore, the correlation coefficient we are looking for is defined as follows

### **Definition 1**

The mapping from $\mathbb {R}^{M\times {N_{1}}}\times \mathbb {R}^{M\times {N_{2}}}$ to [0, 1] is a correlation coefficient if the index satisfies C1 to C4

$$\begin{array}{*{20}l} \begin{aligned} &C1: corr(p*A, B) = corr(A, q*B) = corr(A, B)\\ &C2: corr(A, B) = corr(B, A)\\ &C3: corr(A, B) = 1 \quad if \quad A = f(B)\\ &C4: corr(A, B) = 0 \quad iff \quad A^{T}WB = 0. \end{aligned} \end{array} $$

where *p* and *q* are nonzero scalars and *f*(·) is nonlinear functions acting on the elements of matrix *B*. To overcome difficulties of RV_2_ in detecting nonlinear relationship in C3, we apply a kernel transformation to the matrix *A* and matrix *B* since we can obtain transformed RV-coefficients, RV_*A*_ and RV_*B*_, respectively.

4 $$ RV_{A}(A,B) = \frac{tr\left(\widetilde{f(A)f\left(A^{T}\right)}\widetilde{BB^{T}}\right)}{\sqrt{tr\left[\left(\widetilde{f(A)f\left(A^{T}\right)}\right)^{2}\right]tr\left[\left(\widetilde{BB^{T}}\right)^{2}\right]}}  $$

5 $$ RV_{B}(A,B) = \frac{tr\left(\left(\widetilde{AA^{T}}\right)\widetilde{f(B)f\left(B^{T}\right)}\right)}{\sqrt{tr\left[\left(\widetilde{AA^{T}}\right)^{2}\right]tr\left[\left(\widetilde{f(B)f\left(B^{T}\right)}\right)^{2}\right]}}  $$

where

$$ f(X)=\frac{1}{1+e^{-X}}  $$

$$ f\left(X^{T}\right)=\frac{1}{1+e^{-X^{T}}}.  $$

In order to measure both linear and nonlinear relationships between two matrices, we combine RV_2_ with RV_*A*_ and RV_*B*_ to obtain a new index, i.e., a kernel-based RV-coefficient (KBRV)

6 $$ KBRV(A,B)=\frac{k_{1}}{k_{1}+k_{2}}RV_{2}+\frac{k_{2}}{k_{1}+k_{2}}\frac{RV_{A}+RV_{B}}{2}   $$

where $\frac {k_{1}}{k_{1}+k_{2}}$ and $\frac {k_{2}}{k_{1}+k_{2}}$ are weight coefficients. We can also rewrite the KBRV as

7 $$ KBRV(A,B)=\alpha RV_{2}+(1-\alpha)\frac{RV_{A}+RV_{B}}{2}.   $$

In Eq. (7), the first term measures the linear correlations and the second term measures the nonlinear correlations. Thereby, RV_2_ is a special case of KBRV when *α*=1 while $\rm \frac {RV_{A}+RV_{B}}{2}$ is another case of KBRV as *α*=0. Furthermore, KBRV has a good property since *KBRV*(*A*,*B*) equals *KBRV*(*B*,*A*), which means it is a symmetric index. For analogue data knowing its correlation detail, we could easily choose the most appropriate *α*. However, when it comes to real data, the choice of *α* is particularly important if we want to decide the type and size of the correlation. Here, we use the following formula to determine the optimal *α* for given *A* and *B*

8 $$ \hat{\alpha} = \underset{\alpha}{\arg\max}\,\,KBRV(A,B),   $$

where *α*∈[0,1]. We could estimate the type of correlation based on *α*. For simplicity, when *α*≥0.5, we think the correlation between matrix *A* and *B* is linearly dominant, and we consider it as nonlinearly dominant when *α*<0.5.

To evaluate the effectiveness of KBRV, we applied a permutation test to assess significance of this index. *KBRV*(*A*,*B*) was compared to $KBRV\left (A_{perm_{i}}, B\right)$ in every permutation, where $A_{perm_{i}}$ is the matrix after shuffling the rows and columns of *A* in the *i*th permutation. Subsequently, the *P*-value could be obtained from the following equation

9 $$ p=\frac{{\sum\nolimits}_{i=1}^{n} \mathbb I_{KBRV(A,B)\leq KBRV(A_{perm_{i}},B)}}{n},   $$

where $\mathbb I$ is the indicator function and *n* is the number of permutations. Because RV_2_ is a special case of KBRV (*α*=1), we could compare RV_2_ with KBRV, by the way, when we calculate the significance with different *α*.

Combining Eqs. (8) and (9), the optimal solution $\hat {\alpha }$ is derived from $\underset {\alpha }{\arg \max }\,KBRV(A,B)$ under the condition *p*<*α*_*sig*_, where *α*_*sig*_ equals 0.05 in our research.

### Simulation study

#### Matrix simulation

We generated two matrices *A*(*a*_*ij*_) and *B*(*b*_*ij*_) in several different cases. In the fisrt case, *A*(*a*_*ij*_) and *B*(*b*_*ij*_) were both random matrices drawn from random numbers between 0 and 1, respectively, which means *A* and *B* were independent matrices. In the following cases, *A*(*a*_*ij*_) was drawn in the same way while *b*_*ij*_ in matrix *B* were set as different functions of *a*_*ij*_. Here, noise was derived from standard normal distribution, gamma distribution and bimodal distribution and their levels was discussed [18]. We took the number of rows and columns as 500 and 800, respectively, and different functions (linear, quadratic, sine, exponential, etc.) applying on *b*_*ij*_ with different noise types were explored in Tables 1, 2 and 3. The significance of the permutation test was calculated after 100 permutations. Furthermore, the permutation test was conducted 100 times to calculate the false positive rate/statistical power for independent and dependent matrices, respectively. These simulations are all done on different *α* of KBRV (0, 0.3, 0.5, 0.7, 1, respectively). The computational cost of the permutation test and statistical power are shown in Tables 4–5 (All experiments are executed on an Intel Core i7-8700 running at 3.20 GHz and 16.0 GB memory).

**Table 1:** Power/False negative rate in different functions with Gaussian noise (The significance level *α*=0.05)

*A*∼*Random*(0,1)	*α*=0	*α*=0.3	*α*=0.5	*α*=0.7	*α*=1
*B*∼*Random*(0,1)	0.06	0.07	0.04	0.07	0.06
*B*=*A*+0.5*N*(0,1)	0.92	0.99	1	0.99	1
*B*=(*A*+1)^2^+0.5*N*(0,1)	0.97	1	0.99	1	1
*B*= sin(*A*)+0.5*N*(0,1)	0.90	0.98	0.98	0.98	0.99
*B*= exp(*A*)+0.5*N*(0,1)	0.83	0.96	0.99	1	1

**Table 2:** Power/False negative rate in different functions with Gamma noise (The significance level *α*=0.05)

*A*∼*Random*(0,1)	*α*=0	*α*=0.3	*α*=0.5	*α*=0.7	*α*=1
*B*=*A*+0.5*N*(0,1)	0.79	0.96	0.99	0.97	0.99
*B*=(*A*+1)^2^+0.5*N*(0,1)	0.80	1	0.99	1	1
*B*= sin(*A*)+0.5*N*(0,1)	0.63	0.87	0.92	0.96	0.94
*B*= exp(*A*)+0.5*N*(0,1)	0.63	0.89	0.95	0.96	0.97

**Table 3:** Power/False negative rate in different functions with Bimodal noise (The significance level *α*=0.05)

*A*∼*Random*(0,1)	*α*=0	*α*=0.3	*α*=0.5	*α*=0.7	*α*=1
*B*=*A*+0.3*N*(0,1)	0.77	0.91	0.95	0.98	0.98
*B*=(*A*+1)^2^+0.3*N*(0,1)	0.83	1	1	1	1
*B*= sin(*A*)+0.3*N*(0,1)	0.6	0.84	0.92	0.94	0.99
*B*= exp(*A*)+0.3*N*(0,1)	0.57	0.84	0.93	1	1

**Table 4:** Computational cost of permutation test with different matrix sizes (rows and columns) and number of repetition (permutation)

(*r*,*c*,*n*_*per*_)	(50, 80, 100)	(50, 80, 1000)	(500, 800, 100)	(500, 800, 1000)
Time (seconds)	0.0276	0.2275	2.8792	29.1308

**Table 5:** Computational cost of statistical power with different matrix sizes (rows and columns) and number of repetition (permutation and power)

(*r*,*c*,*n*_*per*_,*n*_*pw*_)	(50, 80, 100, 100)	(50, 80, 1000, 100)	(50, 80, 100, 1000)
Time (seconds)	3.2112	32.8902	32.4776
(*r*,*c*,*n*_*per*_,*n*_*pw*_)	(500, 800, 100, 100)	(500, 800, 1000, 100)	(500, 800, 100, 1000)
Time (seconds)	377.9874	3687.0232	3750.5515

To further validate the rationality of KBRV, we did some simulations concerning matrix size and sparsity. In the first case, we would like to show that as long as two matrices are independent, KBRV is equal to 0 regardless of the size of the matrices. Here, matrix *A*(*a*_*ij*_) of size *M*×500 and *B*(*b*_*ij*_) of size *M*×800 are generated randomly. Under this circumstance, *A* and *B* are repeated 100 times for each *M*, where *M* was the number of matrix’s rows and increased from 50 to 1000 with step size 50. In the second case, *A*(*a*_*ij*_) and *B*(*b*_*ij*_) are drawn from standard normal distribution while a random proportion of elements in *B*(*b*_*ij*_) is replaced by *a*_*ij*_ and $a_{ij}^{2}$ in steps of 10%, 20%, …, and 90%, respectively. These further verifications are all simulated with different *α* of KBRV (0, 0.3, 0.5, 0.7, 1, respectively).

#### Exon-level simulation

To verify the validity of KBRV not only for general matrices, but also for biological data, in this section, we applied KBRV method to exon-level simulation data. Compared to gene expression in vector form, exon-specific expression could be represented as a matrix of order *S*×*J*, where *S* is the number of samples and *J* is the number of exons. Here, we evaluate the performance of KBRV by ROC curves and AUC values based on simulated exon-level gene co-expression networks [19]. For non co-expression genes, we generate two independent *S*×*J*_1/2_ matrices $A_{1}=\left (a_{11},\cdots,a_{1J_{1}}\right)$ and $A_{2}=\left (a_{21},\cdots,a_{2J_{2}}\right)$ which are drawn from a multivariate normal distribution *N*(0,*I*) with $a_{ij}=\left (a_{ij}^{1},\cdots,a_{ij}^{S}\right)^{T}$, for *i*∈{1,2} and *j*∈{1,⋯,*J*_1_} or {1,⋯,*J*_2_}. In contrast with independent gene pairs *A*=(*A*_1_,*A*_2_), dependent (co-expressed) gene pairs *B*=(*B*_1_,*B*_2_) are drawn such that

10 $$\begin{array}{*{20}l} \begin{aligned} &B_{i}=A_{i}+c_{0}F\left(A_{m}^{J_{i}}\right),i=1,2,\\ &A_{m}^{J_{i}}=\left\{\begin{array}{ccc} A_{1}^{J_{i}}=(a_{11},\cdots,a_{1J_{i}}), {J_{1}\geq{J_{2}},}\\ A_{2}^{J_{i}}=(a_{11},\cdots,a_{2J_{i}}), {J_{1}<J_{2}.} \end{array}\right.  \end{aligned} \end{array} $$

where

- *c*_0_ is a constant that indicates the association strength of gene isoforms *B*_1_,*B*_2_.
- $F\left (A_{m}^{J_{i}}\right)$ could be a linear or nonlinear function of $A_{m}^{J_{i}}$.

Here, gene pairs are independent when *c*_0_=0, which means the correlation between *A*_1_ and *A*_2_ should be zero. Meanwhile, *B*_1_ and *B*_2_ are correlated because *c*_0_≠0 bigger *c*_0_ implying stronger correlation. Furthermore, $F\left (A_{m}^{J_{i}}\right)=A_{m}^{J_{i}}$ means a linear relationship between *B*_1_ and *B*_2_ while other nonlinear *F* indicate nonlinear relationships between *B*_1_ and *B*_2_. In our simulations, the number of exons (*k* and *l*) are set to 50-50, 100-100, 50-200. The sample size *S* and the association strength are set to be 50, 100, 200 and 0.1, 0.2, 0.3, respectively. Firstly, as for these 27 combinations, we generate 1000 pairs of matrices (*B*_1_,*B*_2_) under each combination and another 1000 pairs of matrices (*A*_1_,*A*_2_) with the same number of exons and sample size while setting association strength equal to zero. Once having the label of gene pairs, we could use ROC curves and AUC values to evaluate the capabilities of KBRV under different parameters. The effects of the number of exons, sample size and association strength on the KBRV in linear and nonlinear scenarios were discussed respectively. Furthermore, we test KBRV’s ability to distinguish between linear gene pairs and nonlinear gene pairs. We generate 1000 pairs of matrices (*B*_1*l*_,*B*_2*l*_) which are linearly dependent under these 27 combinations and another 1000 pairs of matrices (*B*_1*n*_,*B*_2*n*_) having nonlinear relationships with the same number of exons, sample size and association strength. $F\left (A_{m}^{J_{i}}\right)$ in *B*_1*l*_ and *B*_2*l*_ are set to be $A_{m}^{J_{1}}$ and $A_{m}^{J_{2}}$ while $F\left (A_{m}^{J_{i}}\right)$ in *B*_1*n*_ and *B*_2*n*_ are set to be $A_{m}^{J_{1}}$ and any nonlinear function of $A_{m}^{J_{2}}$, respectively.

### Applications on real datasets

#### Multiomics data

We applied KBRV to ovarian cancer data which are taken from the TCGA database (https://portal.gdc.cancer.gov/repository). This cancer data taken from 385 patients consist of two parts: the gene expression data and the DNA methylation data (The methylation sites within the same gene segement are added up. To keep the multiomics data at the same level, we normalized the gene expression data to [0, 1]). According to previous research, FOXM1 transcription factor network is altered in most ovarian cancer patients [20]. In this network, FOXM1 and its target gene are involved in cell cycle progression and DNA damage repair. Thereby, we picked 9 common genes in both gene expression data and DNA methylation data that are activated in FOXM1 pathway from the total 16406 genes. Firstly, we used vector method, Maximum Information Coefficient (MIC, *α*=0.6), to calculate the correlation between different genes through gene expression data and construct a gene regulatory network. After that, the matrix method, KBRV, was used to calculate the correlation between the nine 385×2 matrices by integrating gene expression data and DNA methylation data. It is worth mentioning that for each calculation, we chose different *α* and combined KBRV value with permutation test, which means the $\hat {\alpha }$ was determined by $\underset {\alpha }{\arg \max }\,KBRV(A,B)$ together with *p*<0.05. After getting the correlation matrix, regulatory edges were selected by introducing a hard threshold. The top 20% are kept in the network.

#### Exon-level data

We further applied KBRV to construct gene regulatory network with the exon-level RNA-Seq data from macrophage, neutrophil and monocyte cell lines in human myeloid differentiation, respectively. Compared with gene-level RNA-Seq data, exon-level RNA-Seq reveals more biological details about gene expression, which in turn requires us to use more advanced methods to calculate because gene samples at the exon-level could be represent as matrices rather than vectors. Here, we focus on 18 transcription factors that are important in human myeloid differentiation [21]. The exon-level data were obtained through DEXSeq package and both exon-level and gene-level data were log-transformed. Correlations between the 18 gene vectors(1×21 samples) and 18 gene matrices(*J*×21 samples) were calculated in three different cell lines using MIC(*α*=0.6) and KBRV($\hat {\alpha }$ was determined by both $\underset {\alpha }{\arg \max }\,KBRV(A,B)$ and *p*<0.05), respectively. Similar to the previous part, top 10% regulatory edges were kept in the network after getting the correlation matrix.

## Results

### Results from simulation study

**Fig. 4:**
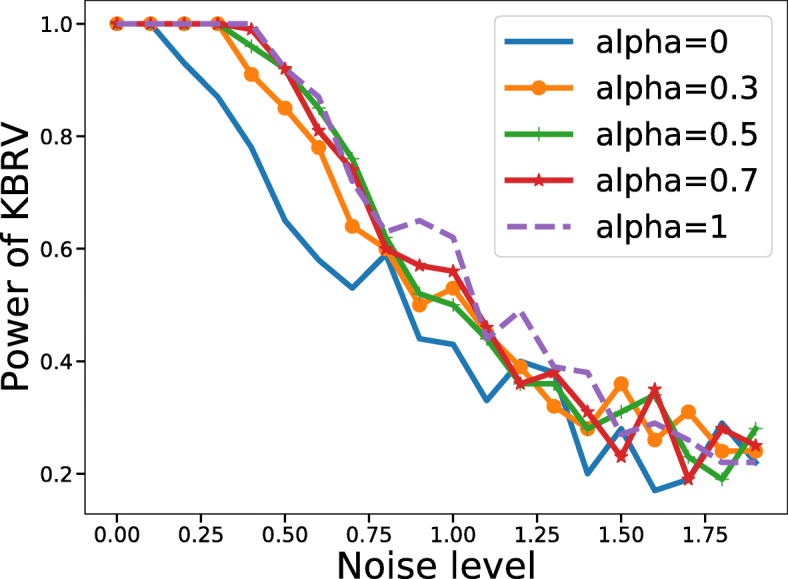
Changes in power with the increase of noise. The curve of statistical power pf KBRV with an increasing gamma noise. The correlation between two matrices is a sine function

The first case in Tables 1, 2 and 3 show that KBRV has only nearly 0.05 of the probability to arrive at the wrong conclusion, that is, correlated, in the case where *A* and *B* are both random matrices. When *A* and *B* are linearly correlated, Tables 1, 2 and 3 demonstrate that KBRV with different *α* could both make good judgements with different types of noise. The remaining rows in Tables 1, 2 and 3 show that KBRV is equally valid for nonlinear relationships. By the way, we increased the noise level from 0 to 2 ×Ga(1, 1). In Fig. 4, all power values decrease with the increase of noise, which confirms that our method is reasonable rather than having a high false positive rate. In calculating significance and statistical power, we have repeated each experiment 100/1000 times for each condition (Tables 4–5). In order to get more accurate results, we recommend more experiments be conducted. However, when the matrix size is large, it takes a lot of time to calculate the statistical power.

**Fig. 5:**
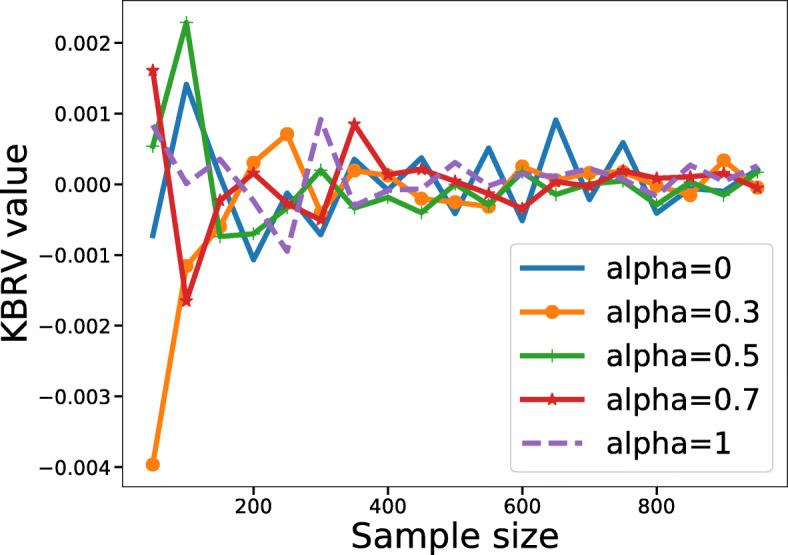
Independent matrices pair of different sizes. The curve of KBRV of 100 repeated tests with an increasing number of rows between two independent matrices *A* and *B*

**Fig. 6:**
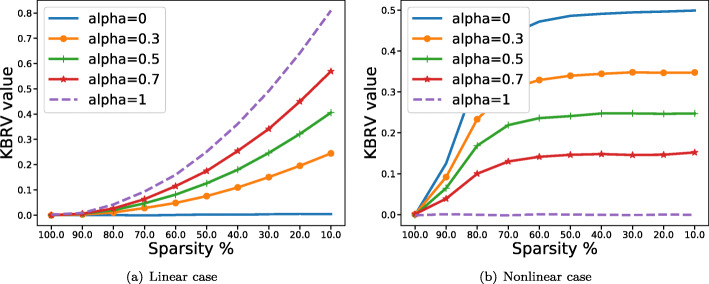
Increasing linear and nonlinear correlations. Comparisons between different indexes of KBRV when the elements of two matrices *A* and *B* are set to be **a** *b*_*ij*_=*a*_*ij*_ and **b**$b_{ij}=a_{ij}^{2}$, respectively. A reverse trend of KBRV’s value is appeared when *α* takes different values in these two cases

From Fig. 5, we could observe that KBRV are almost zero no matter what size matrix *A* and *B* is, indicating they are independent. Besides, it is worth mentioning that the KBRV value fluctuates slightly when sample size is very small, possibly due to the huge difference in the number of rows and columns. The calculated results reflecting sparsity with different parameter *α* are shown in Fig. 6. As we can see, KBRV (*α*=1) has the whole RV_2_ while containing no RV_*A*_ or RV_*B*_, so it predicts the linear relationship best, which appears larger value in Fig. 6a and smaller value in Fig. 6b. With the RV_2_ component decreasing and the RV_*A*_ plus RV_*B*_ part growing, KBRV is losing its power to detect the linear relationship. KBRV (*α*=0) has the whole RV_*A*_ and RV_*B*_ while containing no RV_2_, so it performs better in assessing the nonlinear relationship between the matrices *A* and *B*, which appears larger value in Fig. 6b and smaller value in Fig. 6a. These results demonstrate that KBRV can efficiently evaluate the correlations between two matrices in both the linear and nonlinear case.

**Fig. 7:**
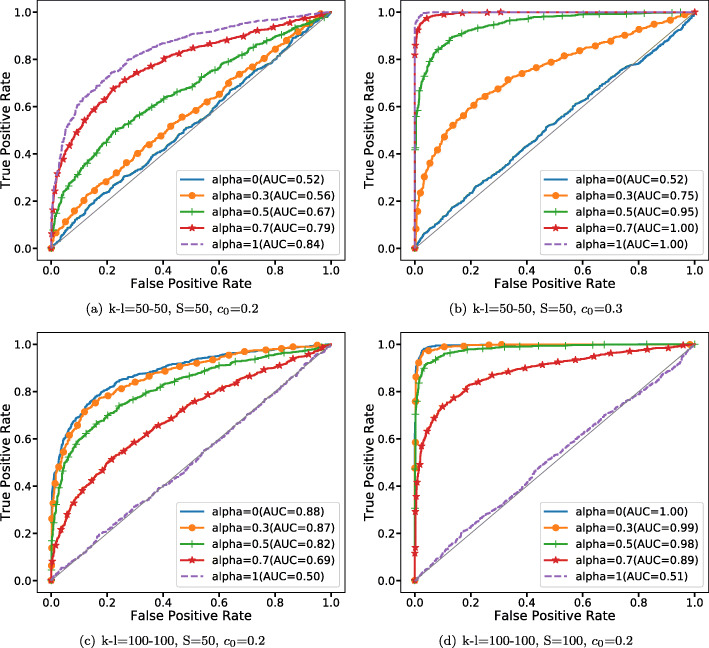
ROC of KBRV in differing independent and dependent exon-level gene network. **a**-**b** The effect of the association strength *c*_0_ on the performances of different KBRV under independent and linear dependent relationship. **c**-**d** The effect of sample size on the performances of different KBRV under independent and cosine dependent relationship

Figure 7a and b reveal the simulation results of the setting of two linear correlated gene pairs with 50-50 exons and sample size equals 50. Under this circumstance, it is observed that AUCs of different KBRV for different *α* were significantly increasing as the association strength *c*_0_ growing except KBRV with *α*=0. However, RV_2_ is a special case of KBRV with *α*=1, which performs better than others. The results for the nonlinear case (cosine) are shown in Fig. 7c and d. We set the numbers of two exons to 100-100, the association strength *c*_0_=0.2 and the sample size from 50 to 100. Figure 7c and d show an increasing trend of KBRV as the sample size increase from 50 to 100 for all methods except KBRV with *α*=1, which is just the opposite of Fig. 7a and b. Based on ROC curves and AUC values, we observe that KBRV (*α*=1) outperforms others when $F\left (A_{m}^{J_{i}}\right)$ is a linear function of $A_{m}^{J_{i}}$ and KBRV (*α*=0) performs the best when $F\left (A_{m}^{J_{i}}\right)$ is a nonlinear function of $A_{m}^{J_{i}}$.

**Fig. 8:**
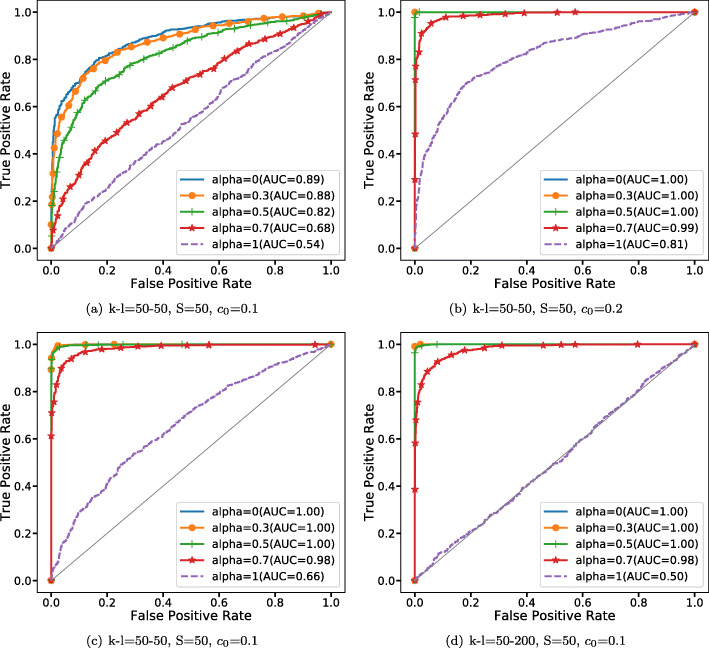
ROC of KBRV in differing linear dependent and nonlinear dependent exon-level gene network. **a**-**b** The effect of the association strength *c*_0_ on the performances of different KBRV under linear dependent and cosine dependent relationship. **c**-**d** The effect of number of exons on the performances of different KBRV under linear dependent and exponential dependent relationship

Figure 7 shows the ability of KBRV in distinguishing independence and dependence. Next we will show KBRV’s ability in distinguishing linear dependence and nonlinear dependence. Figure 8a and b evaluate the effect of KBRV in distinguishing linear and cosine relationships. Here, we set the number of exons to 50-50, sample sizes equals 50 and the association strengths from 0.1 to 0.2. Under these conditions, Fig. 8a and b demonstrate that AUCs of KBRV are all significantly increasing as the association strength *c*_0_ is growing. Most importantly, KBRV is better than RV_2_ (*α*=1) especially when *c*_0_=0.2 where KBRV’s AUC attains 1. Figure 8c–d evaluate the effect of the number of exons on the performance of KBRV when $F\left (A_{m}^{J_{i}}\right)=exp\left (A_{m}^{J_{i}}\right)$. Here, the sample sizes of two exon-level genes are 50 and the association strength *c*_0_=0.1 while the number of exons change from 50-50 to 50-200. From (c) and (d), RV_2_ (*α*=1) becomes smaller as the difference between the number of exons increases. However, KBRV(*α*≠1) continues to maintain high accuracy.

### Results from multiomics data

**Fig. 9:**
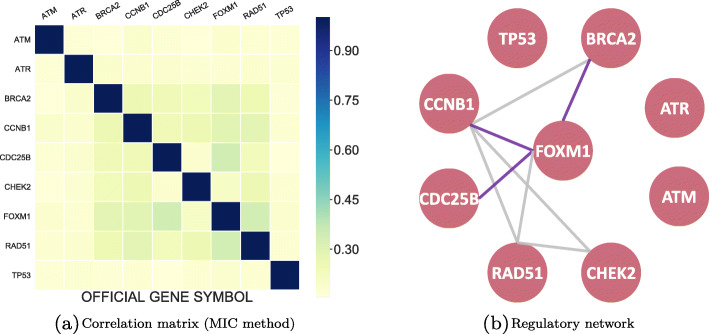
Heat map and its corresponding regulatory network (MIC). **a** Correlation heat map constructed by using gene expression data of ovarian cancer with MIC method. **b** Gene regulatory network obtained from **a**. The solid lines represent inferred transcriptional regulations while the purple lines are regulations have been proven to exist

**Fig. 10:**
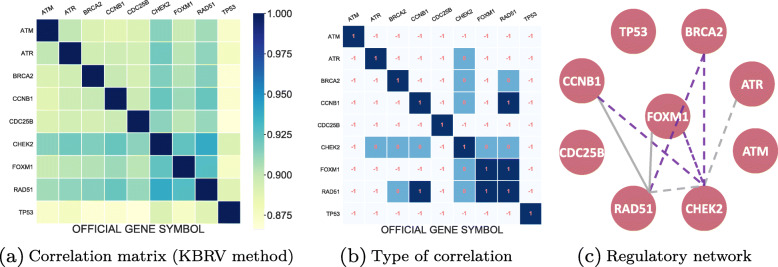
Heat map and correlation type and their corresponding regulatory network (KBRV). **a** Correlation heat map and its correlation type constructed by combining gene expression data and DNA methylation data of ovarian cancer with KBRV method. **b** Gene regulatory network obtained from **a**. The solid lines represent inferred linear transcriptional regulations while the dashed lines indicate nonlinear regulate. Proven regulatory relationships are highlighted in purple

For a heat map of the correlation matrix calculated through MIC shown in Fig. 9a, we observed that the correlations between all genes were weak. But in fact, these genes are highly correlated in patients with ovarian cancer. To make the result more vivid, we turned this heat map into a gene regulatory network with a hard threshold in Fig. 9b. The solid lines are used to represent transcriptional regulations we inferred while the purple lines are regulations have been proven to exist. Instead of using MIC method to calculate vector’s correlation, we applied KBRV method to this integrated gene matrix and used a heat map to display our new gene regulatory networks in Fig. 10. From Fig. 10a, it can be seen that nearly all the calculated correlations are high, which is consistent with the fact. In addition, the advantage of KBRV is that we can infer the type of correlation(linear or nonlinear) from $\hat {\alpha }$. We recorded the optimal $\hat {\alpha }$ corresponding to Fig. 10a after selecting the top 20% regulatory edges (Fig. 10b: 1 corresponds to $\hat {\alpha }=1$, 0 corresponds to $\hat {\alpha }=0$, -1 corresponds to not correlated). With the information in Fig. 10b, we can easily construct the regulatory network. In Fig. 10c, solid lines represent linear regulate while dashed lines indicate nonlinear regulate. Meanwhile, proven regulatory relationships are highlighted in purple. As can be seen from the above comparison, the KBRV method is significantly superior to MIC method. Firstly, the integrated gene regulatory network is more reasonable for its high correlations compared to MIC’s results. Besides, KBRV identifies more confirmed regulatory relationships. Most importantly, KBRV measures the correlation while also identifying the type of correlation, which is not possible with other methods. Furthermore, we consider the nonlinear correlation an indication of indirect correlation. Indirect relationship might appear as nonlinear correlations because the transfer of multiple linear relationships might be nonlinear. For example, in the latter network we built, a nonlinear relationship between BRCA2 and CHEK2 is detected by KBRV as BRCA2 is indirectly regulated by CHEK2 through FOXM1. What’s more, a novel nonlinear regulatory pathway between CHEK2 and ATR is identified. To sum up, the KBRV method integrated the information of two data and surmount the defects of a single data and vector method.

### Results from exon-level data

**Fig. 11:**
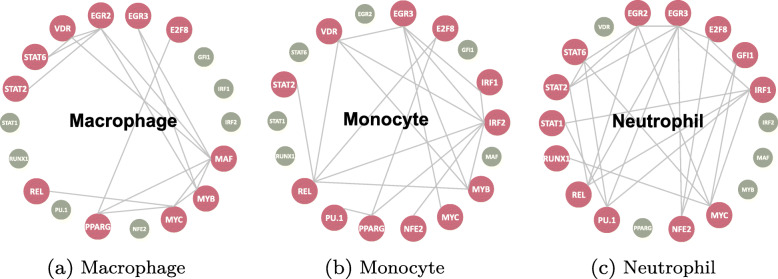
Regulatory networks obtained from MIC method. Regulatory networks obtained from gene-level RNA-Seq data from macrophage **a**, neutrophil **b** and monocyte **c** cell lines in human myeloid differentiation. The solid lines represent inferred transcriptional regulations

**Fig. 12:**
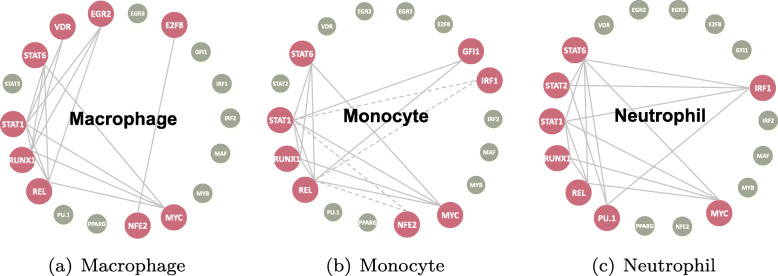
Regulatory networks obtained from KBRV method. Regulatory networks obtained from exon-level RNA-Seq data from macrophage **a**, neutrophil **b** and monocyte **c** cell lines in human myeloid differentiation. The solid lines represent inferred transcriptional regulations while the dashed lines indicate nonlinear regulate

RUNX1, REL and STAT family are the most important genes in human myeloid differentiation. For example, RUNX1 plays a central role in hematopoiesis of all lineages [22]. REL plays a critical part in inflammation, immunity, cell proliferation, differentiation, and survival [23]. STAT1 and STAT6 are important transcription factors that mediate cellular immunity, proliferation, apoptosis and differentiation [24]. The regulatory networks obtained from gene-level vectors are quite different from those drawn from exon-level matrices. Figure 11 demonstrates the network constructed by MIC in three different cell lines. It is hard to say that the networks in (a), (b) and (c) are reasonable because the three networks have nearly nothing in common. In contrast, the networks built through KBRV based on exon-level data is more reasonable and enlightening. Figure 12a-c share MYC, RUNX1, REL, STAT1 and STAT6 and their edges in common, which means these genes and regulatory relationships might be shared functional modules of the three cell lines. Besides, these genes play key roles in human myeloid differentiation as we mentioned before. E2F8, EGR2 and VDR are unique genes involved in the Macrophage cell line, while GFI1 plays a role in the Monocyte cell line. Besides, PU.1 and STAT2 have unique regulatory roles in the Neutrophil cell line. Unlike outcomes in the ovarian cancer study, most of the regulatory relationships here are linear, only IRF1 and NFE2 in Monocyte cell line show nonlinear regulations.

## Discussion

In this study, we proposed a novel index to detect linear and nonlinear correlations for high dimensional data. The proposed KBRV is a universal index, which overcomes the shortcoming that some measures are not able to identify a nonlinear relationship when compared to its special form RV_2_. It is worth noting that KBRV is an extension of RV_2_ and inherited some good properties of RV_2_. We suggest calling them the family of KBRV coefficient. This family of correlation coefficients has a parameter *α*, which regulates its weights for a linear and nonlinear relationship. For simulation data, *α*=1 has the strongest ability to recognize the linear relationship. Meanwhile, *α*=0 has the highest AUC value when the relationship is nonlinear. When it comes to real data, we recommend choosing a step size and exhausting all the results based on both KBRV and permutation test. After that we can figure out the best $\hat {\alpha }$, and from $\hat {\alpha }$ we can in turn claim the type of correlation. When considering a nonlinear correlation, indirect regulation is also considered as a kind of nonlinear relationship in our research. This method has been applied to simulation data and real data and both results show that this correlation coefficient is reliable. It is worth mentioning that in the first set of real data, we show that multiomics data are more illuminating than single omics data, while in the second set of real data, the exon-level data proved to be more revealing than the gene-level data.

However, although the numerical results of KBRV are good, there are still some challenges. First of all, we only get the rationality of the numerical simulation but lack theoretical proof of this index. Besides, when this approach is used to integrate multiomics data, it is inevitable to face associations and differences between different types of data. For example, RNA-seq gene expression data have various expression units such as TPM, RPKM, FPKM and even raw reads counts. However, the level of DNA methylation is usually expressed by *β* value, which represents the ratio of the methylated bead type intensity to the intensity of combined locus. Considering that multiomics data are different in measurement and are difficult to integrate into a single matrix, we think it is possible to consider integrative analysis of multiomics data as a data pre-processing method to obtain standardized multiomics data matrix. For example, robust network-based analysis provides a novel perspective in modeling regulations between gene expressions, copy number variations, DNA methylation and other omics data [25]. Taking the ovarian cancer data as an example, microRNA data for 385 ovarian cancer patients are available from TCGA. However, we did not use these data because they could not correspond to gene expressions and DNA methylation data as a column in the matrix. If we use robust network-based analysis to determine the regulations between gene expression and microRNA, the information from microRNA data could then be integrated into gene expression data properly.

**Fig. 13:**
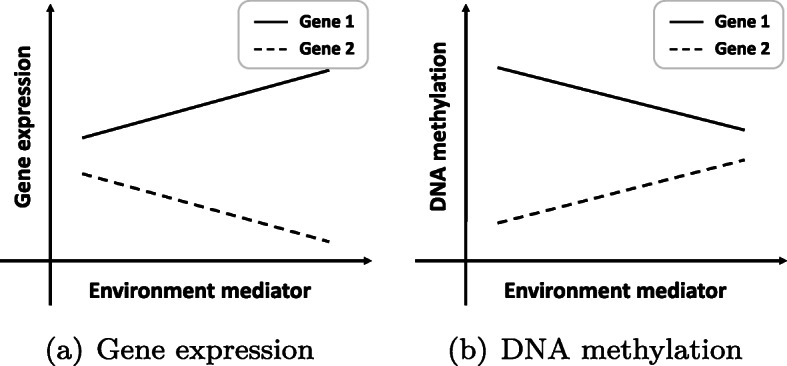
Gene-Environment interactions. Environment variables may have opposite effects on different omics data. **a** Gene expression-Environment interaction **b** DNA methylation-Environment interaction

Appropriate inference about clinical or environmental covariates might help elucidate the genetic basis of complex diseases and improve the accuracy of our method. As far as we know, Wu et al. have proposed many effective methods such as semiparametric bayesian variable selection, additive varying-coefficient model, penalized robust semiparametric approach, etc. in dealing with gene-environment interactions [26–29]. Here, we offer two ideas of combining environmental covariates with genetic data when inferring regulations from the perspective of a matrix. Firstly, the sizes of two matrices are quite small, especially the number of columns. Take the environment into consideration, we can add some columns representing clinical, environmental or phenotype variable to the matrix. These covariates could be discrete or continuous variables, but unified normalization is required because they are listed together with the multiomics data. Secondly, gene-environment interactions might have opposite effects in multiomics data. For example, environmental variables might have a negative effect on gene expression data while exerting a positive effect on DNA methylation data (dashed line in Fig. 13). Therefore, we should consider the different effects of environmental variables on different omics of data when we combine multiomics data into one matrix. By the way, causality research and higher dimensional methods that can integrate multiomics data and exon level data at the same time are also extremely important and have not been involved yet. However, quantifying the concrete form of the nonlinear correlation for biological data using the KBRV framework remains challenging, and it is definitely worth further investigation in the future.

## Conclusions

In this study, we brought up an efficient index for detecting both linear and nonlinear relationships for high dimensional data, named KBRV, which has a broader application scenario than the original index, RV_2_. The KBRV was used to construct regulatory networks after its rationality was verified by simulated matrices and simulated exon-level data. As the dimensions of the data increases, some novel methods have been brought up in order to work out some problems that can not be solved by conventional approaches. As for correlation studies, the expansion from low dimensions to higher dimensions is also indispensable. Today, most existing methods focus on vector data, but there is a lack of research on matrix or even tensor data. Improper use of vector methods could lead to partial or even opposite conclusions. Therefore, correlation methods like KBRV for detecting high dimensional data rather than vector data have potential value in practical application such as the construction of gene regulatory networks, co-expression networks, etc.

## Data Availability

The ovarian cancer dataset were downloaded from the TCGA datasets (https://portal.gdc.cancer.gov/repository).The raw RNA-Seq data from three cell lines in human myeloid differentiation could be abtained from GSE79044. The processed exon-level RNA-Seq data and simulation codes are publicly available from https://github.com/sweetBugs/kbrv-method.

## Abbreviations

RV_2_: The modified RV-coefficient
KBRV: The Kernel-based RV-coefficient
MIC: Maximal information coefficient
TCGA: The cancer genome atlas
ROC: Receiver operating characteristic
AUC: Area under the curve
FOXM1: Forkhead box M1
BRCA2: Breast cancer 2
CHEK2: Checkpoint kinase 2
ATR: Ataxia telangiectasia and Rad3 related
RUNX1: Runt related transcription factor 1
REL: REL Proto-oncogene
STAT1: Signal transducer and activator of transcription 1
STAT2: Signal transducer and activator of transcription 2
STAT6: Signal transducer and activator of transcription 6
MYC: MYC Proto-oncogene
E2F8: E2F transcription factor 8
EGR2: Early growth response 2
VDR: Vitamin D receptor
GFI1: Growth factor independent 1 transcriptional repressor
PU.1: Spi-1 Proto-oncogene
IRF1: Interferon regulatory factor 1
NFE2: Nuclear factor erythroid 2
TPM: Transcripts per Kilobase million
RPKM: Reads per Kilobase million
FPKM: Fragments per Kilobase million
